# *Tobacco vein banding mosaic virus* 6K2 Protein Hijacks NbPsbO1 for Virus Replication

**DOI:** 10.1038/srep43455

**Published:** 2017-02-23

**Authors:** Chao Geng, Zhi-Yong Yan, De-Jie Cheng, Jin Liu, Yan-Ping Tian, Chang-Xiang Zhu, Hong-Yan Wang, Xiang-Dong Li

**Affiliations:** 1 Laboratory of Plant Virology, Department of Plant Pathology, College of Plant Protection, Shandong Agricultural University, Tai’an, Shandong 271018, China; 2 Shandong Provincial Key laboratory for Agricultural Microbiology, Tai’an, Shandong, 271018, China

## Abstract

Chloroplast-bound vesicles are key components in viral replication complexes (VRCs) of potyviruses. The potyviral VRCs are induced by the second 6 kDa protein (6K2) and contain at least viral RNA and nuclear inclusion protein b. To date, no chloroplast protein has been identified to interact with 6K2 and involve in potyvirus replication. In this paper, we showed that the Photosystem II oxygen evolution complex protein of *Nicotiana benthamiana* (NbPsbO1) was a chloroplast protein interacting with 6K2 of *Tobacco vein banding mosaic virus* (TVBMV; genus *Potyvirus*) and present in the VRCs. The first 6 kDa protein (6K1) was recruited to VRCs by 6K2 but had no interaction with NbPSbO1. Knockdown of *NbPsbO1* gene expression in *N. benthamiana* plants through virus-induced gene silencing significantly decreased the accumulation levels of TVBMV and another potyvirus *Potato virus Y*, but not *Potato virus X* of genus *Potexvirus*. Amino acid substitutions in 6K2 that disrupted its interaction with NbPsbO1 also affected the replication of TVBMV. NbPsbP1 and NbPsbQ1, two other components of the Photosystem II oxygen evolution complex had no interaction with 6K2 and no effect on TVBMV replication. To conclude, 6K2 recruits 6K1 to VRCs and hijacks chloroplast protein NbPsbO1 to regulate potyvirus replication.

To achieve successful infection in plant, positive-stranded RNA viruses utilize host cellular membranes to form viral replication complexes (VRCs)^1^,^2^,^3^,^4^. Formation of VRCs can occur on various organelle membranes including endosome, endoplasmic reticulum (ER), golgi membrane, chloroplast membrane, mitochondria membrane, peroxisome and plasma membrane^1^,^2^,^3^,^5^. VRCs were reported to contain viral RNA, viral replication-associated proteins and host proteins like RNA-modifying enzymes, protein chaperones, ESCRT proteins, translation factors and proteins involved in lipid biosynthesis^6^,^7^. Identification of new host factor(s) necessary for virus replication will further advance our understanding of virus replication and the development of novel and sustainable antiviral strategies for agriculture.

Chloroplast is the metabolic energy manufacture in plant. It is also known to have an important role in plant virus replication. For example, *Turnip yellow mosaic virus* 140 K protein contains methyltransferase, proteinase and NTPase/helicase motifs and targets chloroplast envelope. This 140 K protein can recruit the 66 K viral RNA-dependent RNA polymerase (RdRp) to chloroplast periphery^8^,^9^. *Alfalfa mosaic virus* (AMV) and *Barely stripe mosaic virus* (family *Bromoviridae*) also target chloroplast for their replication^10^,^11^,^12^. Several chloroplast proteins have now been identified to interact with viral proteins during virus infection in plant. The replicase of *Tobacco mosaic virus* (TMV) was shown to interact with Rubisco activase (RCA) and silencing *RCA* gene expression through virus-induced gene silencing (VIGS) enhanced TMV accumulation in cells^13^. The movement protein of *Tomato mosaic virus* (ToMV) interacted with Rubisco small subunit (RbCS) and knockdown of *RbCS* expression enhanced ToMV infection in virus-inoculated plant^14^.

Photosystem II (PS II) was reported to function as a light-driven, water-plastoquinone oxidoreductase. In addition, the PS II contains several extrinsic oxygen evolution complex (OEC) proteins that are known to have key roles in stabilizing the active manganese site^15^,^16^. OEC proteins are also known to interact with viral proteins. For example, the PS II OEC protein PsbP was shown to interact with AMV coat protein and *Rice stripe virus* (RSV) disease-specific protein. Transient over-expression of *PsbP* in *N. benthamiana* leaves led to a significant reduction of AMV accumulation. Similarly, over-expression of *PsbP* in rice plant through stable transformation strongly suppressed the accumulation of RSV in RSV-inoculated plants^17^,^18^. *N. benthamiana* PsbO (NbPsbO) interacted with the helicase domain of TMV replicase and the TGBp3 of *Alternanthera mosaic virus*^19^,^20^. Interestingly, silencing *NbPsbO* gene in *N. benthamiana* plants using a *Tobacco rattle virus* (TRV)-based VIGS vector resulted in a 10-fold increase of TMV RNA accumulation^19^. Although replication of many potyviruses was shown to associate with chloroplast^21^,^22^,^23^,^24^,^25^, no chloroplast protein has been reported to interact with potyvirus 6K2 protein, which induces the formation of VRCs.

Potyviruses (genus *Potyvirus,* family *Potyviridae*) are members in the picornarvirus-like superfamily^26^,^27^. They form the largest genus of plant-infecting virus and often cause great economic losses to food crop productions worldwide^28^. Potyvirus genome is a positive sense, single-stranded RNA of approximately 10 kb in length. It contains two open reading frames (ORF) encoding two polyproteins that are later processed into 11 mature proteins by three virus-encoded proteinases^29^. Among the 11 mature proteins, the third protein (P3), cylindrical inclusion (CI), the second 6-kDa protein (6K2), nuclear inclusion protein a (NIa) and nuclear inclusion protein b (NIb) are known to associate with potyviral VRCs^25^,^30^. The 6K2 could induce ER membrane-bound vesicles and could target chloroplast to induce chloroplast invagination^25^. The first 6-kDa protein (6K1) was recently shown to localize inside the VRCs of *Plum pox virus* (PPV)^31^. But how 6K1 is recruited to VRCs and its role in potyvirus infection in plant remains largely unknown.

*Tobacco vein banding mosaic virus* (TVBMV) is a member of genus *Potyvirus* and infects mainly solanaceous plant species^32^,^33^,^34^,^35^,^36^. In this study, we determined that TVBMV 6K1 interacted with 6K2, and was recruited by 6K2 to VRCs found to be adjacent to chloroplast. During TVBMV infection, 6K1 co-localized with the RdRp and 6K2 protein in VRCs. We further showed that 6K2 but not 6K1 interacted with NbPsbO1. Silencing the expression of *NbPsbO1* gene in *N. benthamiana* using a TRV-based VIGS vector decreased the accumulation of TVBMV and *Potato virus Y* (PVY) but not *Potato virus X* (PVX). Also in this study, silencing the expression of *NbPsbP1* or *NbPsbQ1* did not affect the replication of TVBMV. Together, our results indicate that the 6K1 is an essential component in potyviral VRCs and *NbPsbO1* is an important host factor involved in potyvirus replication in plant via interacting with 6K2.

## Results

### Mutations introduced into the RSD motif in 6K1 inhibited TVBMV replication

**Figure 1:**
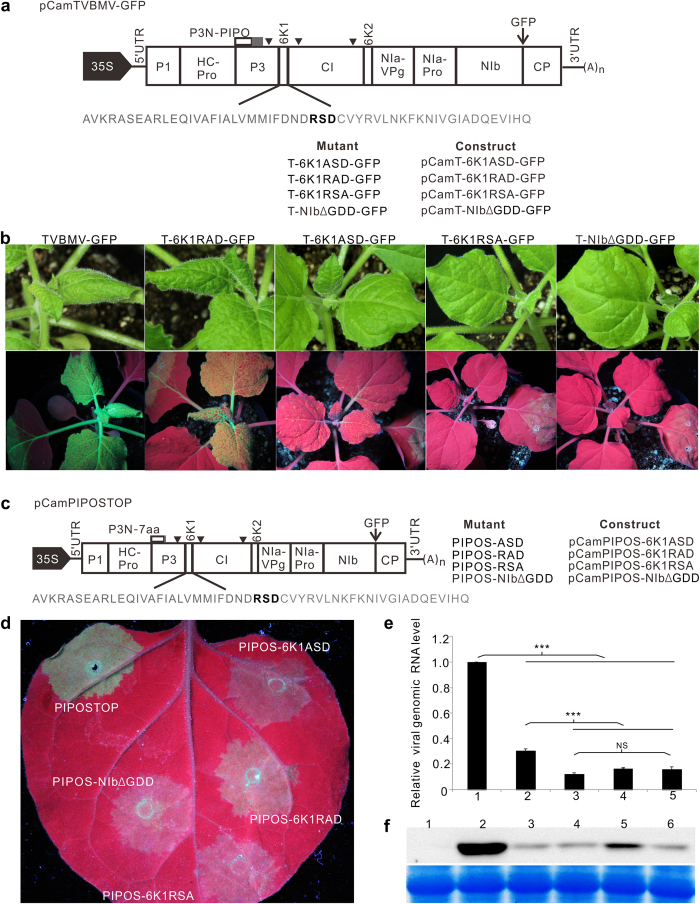
Infectivity of the wild type and mutant TVBMV in *N. benthamiana* plants. (**a**) Genome diagram of pCamTVBMV-GFP is shown at the top. Amino acids (aa) of TVBMV 6K1 are shown in gray. The RSD motif is shown in bold. The names of mutants and constructs were listed below the 6K1 aa sequence to illustrate the mutations introduced into the RSD motif. Arrowheads indicate the positions of the three introns inserted in the pCamTVBMV-GFP clone. (**b**) Phenotypes of the wild type TVBMV-GFP and the four mutant viruses inoculated plants. Images were taken under the normal light (upper panel) or UV illumination (lower panel) at 7 days post agro-infiltration (dpai). (**c**) Schematic representation of pCamPIPOSTOP construct and its mutants with aa changes in the RSD motif. Names of the constructs and mutants are shown. (**d**) GFP fluorescence in the regions infiltrated with various constructs at 4 dpai. (**e**) qRT-PCR analysis of TVBMV genomic RNA accumulations in the PIPOSTOP or its mutants infiltrated *N. benthamiana* leaves at 4 dpai. ***Indicate *P* value < 0.001. 1, PIPOSTOP; 2, PIPOS-RAD; 3, PIPOS-ASD; 4, PIPOS-RSA; 5, PIPOS-NIbΔGDD. (**f**) Western blot assay for TVBMV CP accumulation in the PIPOSTOP or its mutants infiltrated *N. benthamiana* leaves at 4 dpai. 1, Mock; 2, PIPOSTOP; 3, PIPOS-NIbΔGDD; 4, PIPOS-ASD; 5, PIPOS-RAD; 6, PIPOS-RSA.

To determine which region in 6K1 is responsible for TVBMV replication, we obtained 6K1 amino acid sequences of 10 different potyviruses deposited in the GenBank and aligned them. Result showed that 6K1 has a conserved RSD motif in the middle region of the protein (Supplementary Fig. 1). Alanine scanning mutagenesis was then done to modify the RSD motif in the pCamTVBMV-GFP infectious clone produced previously^37^ (Fig. 1a). The wild type (WT) and mutant TVBMV constructs were individually infiltrated into *N. benthamiana* leaves. By 7 days post agro-infiltration (dpai), plants infiltrated with pCamTVBMV-GFP or pCamT-6K1RAD-GFP developed similar mosaic symptoms in upper non-inoculated leaves (systemically infected leaves). Under UV illumination, plants inoculated with TVBMV-GFP showed strong GFP fluorescence in both infiltrated and systemically infected leaves. Plants inoculated with T-6K1RAD-GFP showed a weak GFP fluorescence in both infiltrated and systemically infected leaves (Fig. 1b). The T-6K1ASD-GFP mutant infiltrated plants showed mild epinasty and very weak GFP fluorescence in systemically infected leaves. Plants infiltrated with T-6K1RSA-GFP showed GFP fluorescence only in the infiltrated leaves but failed to produce any virus-like symptoms in systemically infected leaves. Similar result was seen in plants infiltrated with T-NIbΔGDD-GFP, a TVBMV-GFP replication deficient mutant (Fig. 1b).

We then constructed more mutants by replacing the RSD motif with RAD, ASD or RSA in 6K1 of PIPOSTOP, a GFP-tagged TVBMV mutant capable of replicating normally in individual cells but is defective in intercellular movement^37^, or by deleting the GDD motif in NIb of PIPOSTOP to produce pCamPIPOS-6K1ASD, pCamPIPOS-6K1RAD, pCamPIPOS-6K1RSA and pCamPIPOS-NIbΔGDD (Fig. 1c). These mutants were individually infiltrated into *N. benthamiana* leaves. Results of the study showed that green fluorescence in the PIPOSTOP-infiltrated tissues was much stronger than that in other mutant infiltrated tissues (Fig. 1d). Among the four mutants tested, green fluorescence in the PIPOS-6K1RAD-infiltrated tissues was the strongest. No significant difference was observed for the other three mutants (Fig. 1d). Results of qRT-PCR showed that the accumulation level of viral genomic RNA in the PIPOS-6K1RAD-infiltrated tissues was higher than that in the tissues infiltrated with one of the three mutants, but was significantly lower when compared with the parental PIPOSTOP mutant (Fig. 1e). Western blot assay using a TVBMV CP specific antibody agreed with the qRT-PCR results (Fig. 1f).

### TVBMV 6K2 recruited 6K1 to VRCs

**Figure 2:**
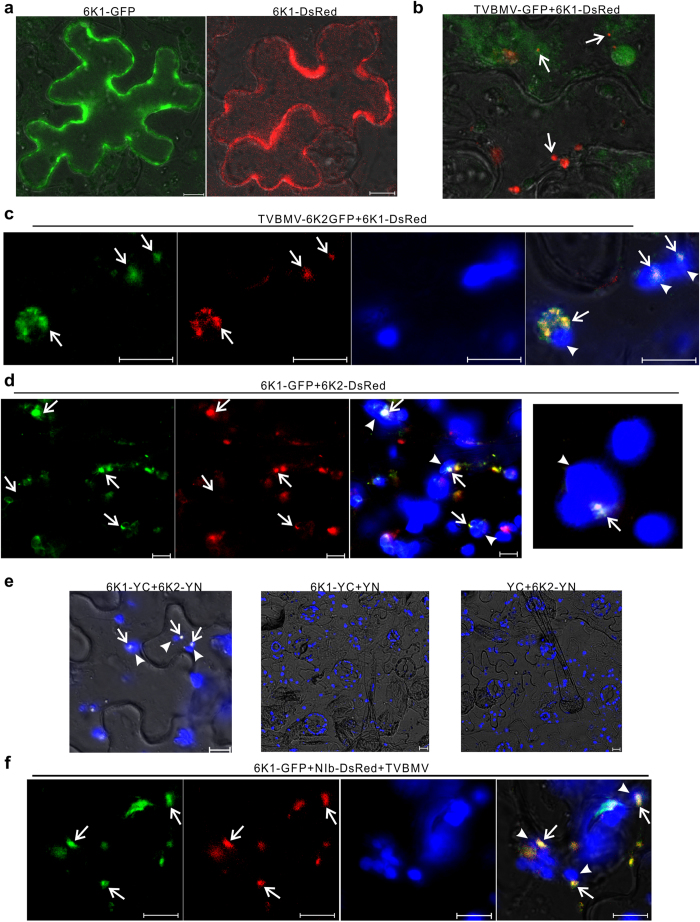
Sub-cellular localization of TVBMV 6K1 and 6K2 in *N. benthamiana* epidermal cells. (**a**) Transient expression of 6K1-GFP or 6K1-DsRed in *N. benthamiana* leaf epidermal cells. (**b**) Localization of 6K1-DsRed in a TVBMV-GFP infected cells. Arrows point to 6K1-DsRed punctate bodies. (**c**) Co-localization of 6K1-DsRed and 6K2-GFP in association with chloroplasts in a TVBMV infected *N. benthamiana* epidermal cell. Images (left to right) show GFP fluorescence, DsRed fluorescence, chloroplast auto-fluorescence, and overlay of the first three images, respectively. Arrows indicate the co-localized fluorescence signal. Arrowheads point to chloroplasts. (**d**) Co-localization of 6K1-GFP and 6K2-DsRed adjacent to chloroplasts. Images are arranged as indicated in (**c**). (**e**) Images of *N. benthamiana* cells co-expressing 6K1-YC and 6K2-YN, 6K1-YC and YN, or YC and 6K2-YN. Arrows point to 6K1–6K2 complexes and arrowheads point to chloroplasts. (**f**) Co-localization of 6K1-GFP and NIb-DsRed in a TVBMV infected *N. benthamiana* epidermal cell. Arrows point to 6K1-GFP or NIb-DsRed punctates and arrowheads point to chloroplasts. Images are arranged as indicated in (**c**). All images were taken at 48 hpai. Scale bars = 10 μm.

To further investigate the role of 6K1 in TVBMV replication, the *6K1* gene was PCR amplified and ligated to the 5′-end of a *GFP* or *DsRed* gene to generate plasmid p6K1-GFP and p6K1-DsRed, respectively. After infiltrating these two constructs individually into *N. benthamiana* leaves, green and red fluorescence were observed freely in the cytoplasm of leaf epidermal cells (Fig. 2a). However, when p6K1-DsRed was infiltrated into TVBMV-GFP infected *N. benthamiana* leaves, red fluorescence from 6K1-DsRed appeared as small punctates (Fig. 2b), resembling the 6K2-induced vesicles reported previously for other potyviruses^25^. To confirm this observation, we co-infiltrated *N. benthamiana* leaves with pCamTVBMV-6K2-GFP, a plasmid expressing all 11 TVBMV encoded proteins plus an additional 6K2-GFP fusion, and p6K1-DsRed. By 48 hours post agro-infiltration (hpai), green fluorescence from 6K2-GFP and red fluorescence from 6K1-DsRed were observed together in punctate structures adjacent to chloroplast in infiltrated cells (Fig. 2c). To determine whether 6K2 alone could modulate the sub-cellular distribution of 6K1, we co-expressed 6K1-GFP and 6K2-DsRed in *N. benthamiana* leaves in the absence of TVBMV infection. Result of the co-infiltration confirmed that yellow fluorescence caused by a co-localization of the two fusion proteins was indeed associated with chloroplast (Fig. 2d). Re-localization of 6K1-GFP to chloroplast in the presence of 6K2-DsRed suggested an interaction between these two proteins. To further confirm this observation, we performed BiFC assays using 6K1-YC (6K1 fused to the C-terminus of YFP) and 6K2-YN (6K2 fused to the N-terminus of YFP). By 48 hpai, yellow fluorescent punctates were observed adjacent to chloroplast (Fig. 2e). No YFP fluorescence was observed in leaf cells co-expressing 6K1-YC and YN, or YC and 6K2-YN (Fig. 2e). To determine if 6K1 co-localizes with viral replicase NIb, the *NIb* gene of TVBMV was PCR amplified and ligated to the *DsRed* gene to generate construct pNIb-DsRed. In the TVBMV infected cells, NIb-DsRed and 6K1-GFP did co-localize adjacent to chloroplast (Fig. 2f). These results further confirmed that TVBMV 6K1 was a component of potyvirus replication complex.

### 6K2 but not 6K1 interacted with NbPsbO1 *in vivo*

To identify host factor(s) present in the 6K1–6K2 containing VRC, we modified pCamTVBMV-GFP to produce pCamTVBMV-6K1GFP. This new construct was confirmed to infect *N. benthamiana* plants systemically and express a free 6K1-GFP fusion (Supplementary Fig. 2). Total protein was extracted from systemically infected leaves of the TVBMV-6K1GFP- or TVBMV-GFP-inoculated plants followed by immunoprecipitation assays using an anti-GFP agarose beads. The co-precipitated proteins were separated in SDS-PAGE gels prior to coomassie blue staining. Several unique protein bands were observed in lanes loaded with total protein from the TVBMV-6K1GFP infected leaves (Data not shown). Liquid chromatography- tandem mass spectrometry was used to characterize the proteins in these unique bands. After database searching, an *N. benthamiana* photosystem II oxygen-evolving complex protein NbPsbO1 was identified with high scores and peptide coverage.

**Figure 3:**
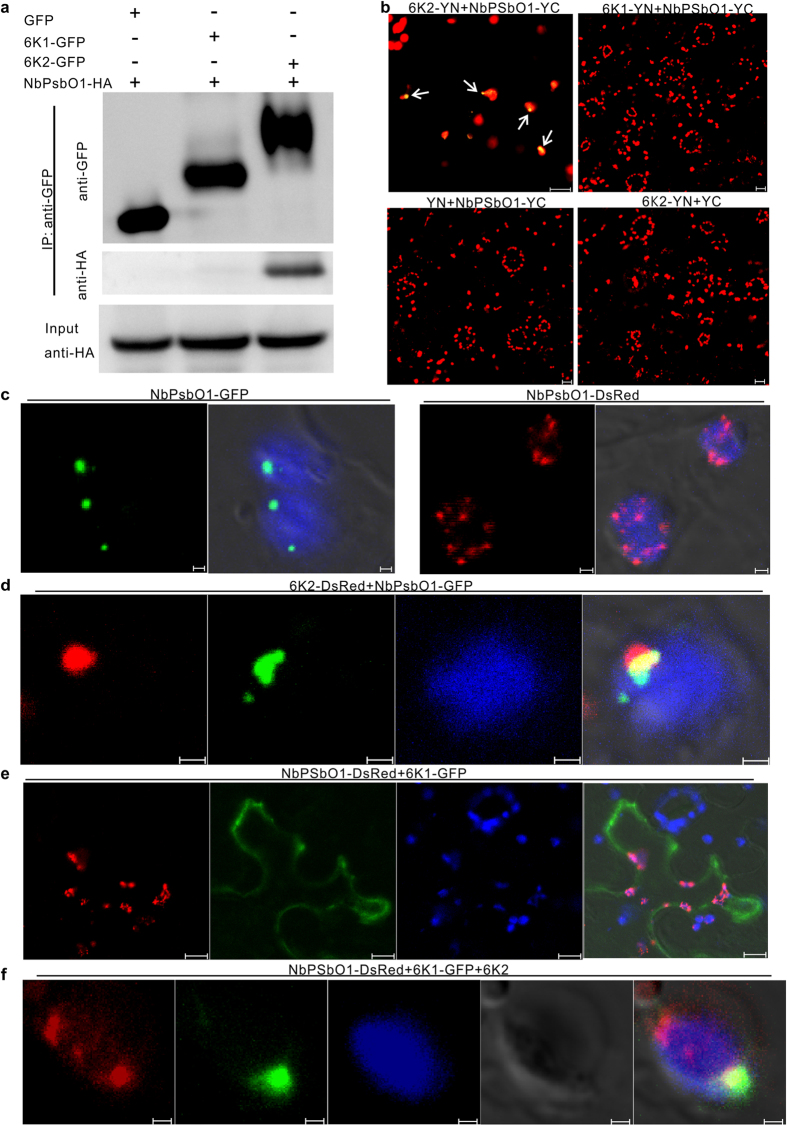
Interaction of 6K2 with NbPsbO1. (**a**) Co-immunoprecipitation (Co-IP) assay for 6K2 and NbPsbO1. 6K2-GFP and NbPsbO1-HA were co-expressed in *N. benthamiana* leaves via agro-infiltration. *N. benthamiana* leaves co-expressing 6K1-GFP and NbPsbO1-HA, or GFP and NbPsbO1-HA were used as negative controls. (**b**) Images of *N. benthamiana* cells co-expressing 6K2-YN and NbPsbO1-YC, 6K1-YN and NbPsbO1-YC, YN and NbPsbO1-YC, 6K2-YN and YC. Punctates containing 6K2-YN and NbPsbO1-YC were indicated with arrows. (**c**) Transient expression of NbPsbO1-GFP and NbPsbO1-DsRed in *N. benthamiana* leaf epidermal cells. Images (left to right) show NbPsbO1-GFP green punctates, overlay of NbPsbO1-GFP green punctates with chloroplast auto-fluorescence, NbPsbO1-DsRed red punctates, and overlay of NbPsbO1-DsRed red punctates with chloroplast auto-fluorescence, respectively. (**d**) Co-localization of 6K2-DsRed and NbPsbO1-GFP in association with a chloroplast. Images (left to right) show 6K2-DsRed red punctate, NbPsbO1-GFP green punctate, chloroplast auto-fluorescence, and overlay of the three images. (**e**) Localization of NbPsbO1-DsRed punctate and 6K1-GFP fluorescence in a cell. Images (left to right) show NbPsbO1-DsRed red punctate, 6K1-GFP fluorescence, chloroplast auto-fluorescence, and overlay of the three images. (**f**) Co-expression of NbPsbO1-DsRed, 6K1-GFP and non-tagged 6K2 in a cell. Images (left to right) show NbPsbO1-DsRed fluorescence, 6K1-GFP fluorescence, chloroplast auto-fluorescence, differential interference contrast and overlay of the four images. All images were taken at 48 hpai. Scale bars in (**c**), (**d** and **f**) equal to 1 μm; Scale bars in others panels equal to 10 μm.

Identification of NbPsbO1 prompted us to investigate the possible interaction between NbPsbO1 and TVBMV 6K1 or 6K2. We first tested the possible interaction between NbPsbO1 and 6K1 or 6K2 via co-immunoprecipitation (Co-IP) assay *in planta*. In this assay, GFP, 6K1-GFP and 6K2-GFP were individually co-expressed with HA-tagged NbPsbO1 in *N. benthamiana* leaves through *Agrobacterium*-mediated infiltration. Total protein was immunoprecipitated using GFP-Trap beads and analyzed by Western blot assay using an HA specific antibody. The results showed that NbPsbO1 was immunoprecipitated with 6K2 but not 6K1 or GFP (Fig. 3a). BiFC assays were then used to further determine the potential interaction between NbPsbO1 and 6K1 or 6K2. Full length NbPsbO1 was fused to the C-terminus of YFP. The resulting plasmid pNbPsbO1-YC was co-infiltrated into *N. benthamiana* leaves with p6K1-YN or p6K2-YN. By 48 hpai, yellow spherules were observed in association with chloroplast in *N. benthamiana* cells co-expressing 6K2-YN and NbPsbO1-YC (Fig. 3b). No YFP fluorescence was detected in cells co-expressing 6K1-YN and NbPsbO1-YC, YN and NbPsbO1-YC, or 6K2-YN and YC (Fig. 3b).

To determine the sub-cellular localization pattern of NbPsbO1 in *N. benthamiana* cells, we fused full length *NbPsbO1* gene to a *GFP* or *DsRed* gene to generate pNbPsbO1-GFP and pNbPsbO1-DsRed, respectively. After infiltration of these constructs to *N. benthamiana* leaves, vesicle-like structures were observed in association with chloroplast by 48 hpai (Fig. 3c). When 6K2-DsRed and NbPsbO1-GFP were co-expressed in *N. benthamiana* leaf cells, red and green fluorescence were observed together in association with chloroplast (Fig. 3d). In contrast, red and green fluorescence from NbPsbO1-DsRed and 6K1-GFP were found separate from each other in cells (Fig. 3e). Because 6K2 could recruit 6K1 to chloroplast, in the presence of 6K2, 6K1-GFP fluorescence was indeed found to overlap with red fluorescence from NbPsbO1-DsRed (Fig. 3f).

### Knockdown of *NbPsbO1* suppressed TVBMV replication

**Figure 4:**
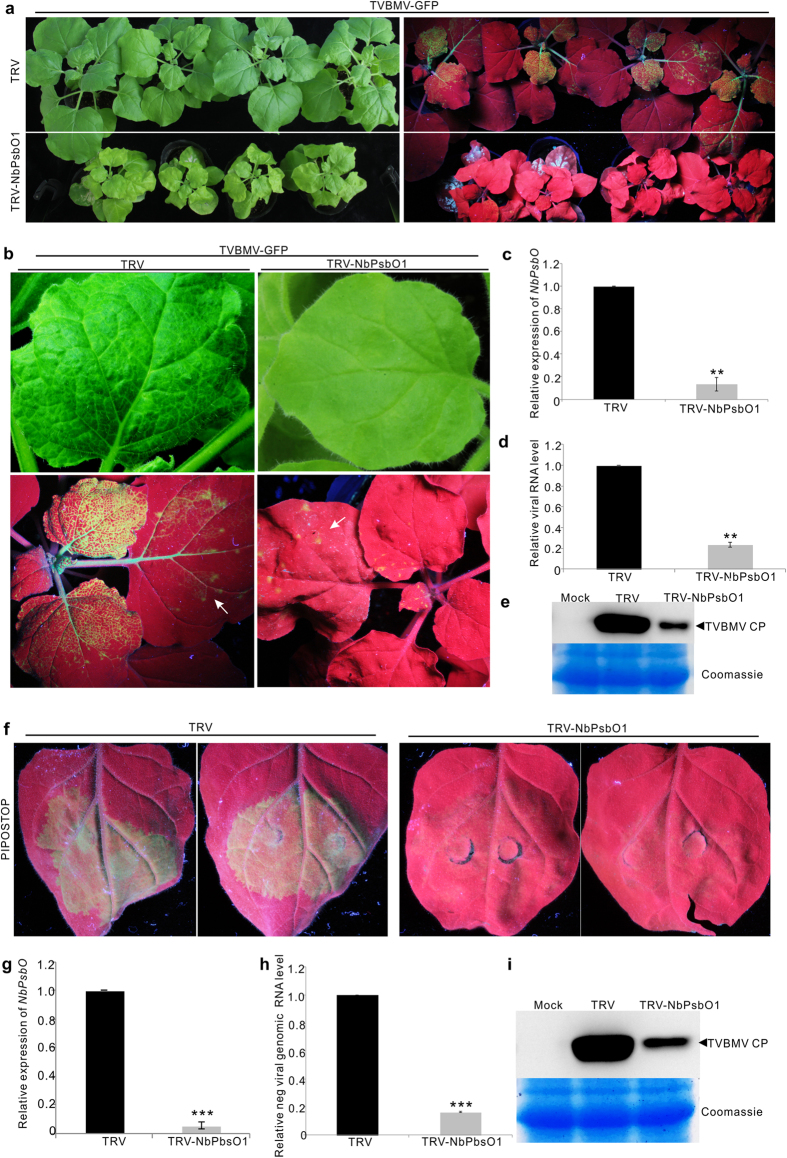
Infectivity of TVBMV-GFP in NbPsbO1-silenced or non-silenced *N. benthamiana* plants. (**a**) TVBMV-GFP inoculated NbPsbO1-silenced (TRV- NbPsbO1) or non-silenced (TRV) control *N. benthamiana* plants (left). Same plants photographed under UV illumination at 6 dpi are shown at right. (**b**) Close up of a TVBMV-GFP inoculated NbPsbO1-silenced (right) and a non-silenced (left) plant at 6 dpi. White arrows indicate the inoculated leaves. (**c**) qRT-PCR analysis for *NbPsbO1* transcript accumulation in the TVBMV-GFP inoculated NbPsbO1-silenced and non-silenced control plants at 6 dpi. The expression level of *EF-1a* gene was used as an internal control. Error bars represent the standard deviations from three biological replicates. Statistical differences were determined by two-sample *t*-test. **Indicates *P* value < 0.01. (**d**) qRT-PCR analysis TVBMV genomic RNA accumulation in the NbPsbO1-silenced or non-silenced control plants. The expression level of *EF-1a* was used as an internal control. Error bars represent standard deviations from three independent biological replicates. **Indicates *P* value < 0.01. (**e**) Immunoblot analysis of TVBMV-GFP CP accumulation in the NbPsbO1-silenced and non-silenced control *N. benthamiana* plants at 6 dpi. Coomassie Blue staining was used to show sample loadings. (**f**) GFP fluorescence in the PIPOSTOP inoculated NbPsbO1-silenced and non-silenced control plants at 4 dpai. (**g**,**h**) qRT-PCR analysis for *NbPsbO1* transcript and TVBMV genomic RNA accumulations in the NbPsbO1-silenced and non-silenced plants. Error bars represent standard deviations from three independent biological replicates. ***Indicate *P* value < 0.001. (**i**) Western blot analysis of PIPOSTOP CP accumulation in the NbPsbO1-silenced and non-silenced control plants. Coomassie Blue staining was used to show sample loadings.

To determine the role of NbPsbO1 in TVBMV infection, a 500-bp fragment was PCR amplified from the *NbPsbO1* gene and cloned into a TRV-based VIGS vector^38^. The *N. benthamiana* plants inoculated with TRV- NbPsbO1 VIGS vector showed mild chlorosis in their leaves and stunting of the plants compared with the control plants infiltrated with an empty TRV VIGS vector (Fig. 4a, left). After the NbPsbO1-silenced or non-silenced control *N. benthamiana* plants were rub-inoculated with TVBMV-GFP, much weaker GFP fluorescence was observed in the NbPsbO1-silenced plants than that in the control plants by 6 days post virus inoculation (dpi) (Fig. 4a, right). At a higher magnification, mosaic symptoms and GFP fluorescence were observed in the systemically infected leaves of the TVBMV-GFP inoculated control *N. benthamiana* plants (Fig. 4b, left). In contrast, no apparent virus symptoms and very week GFP fluorescence were observed in the systemically infected leaves of the NbPsbO1-silenced *N. benthamiana* plants (Fig. 4b, right). Results of qRT-PCR showed that the expression level of *NbPsbO1* in NbPsbO1-silenced plants was only about 14% of that in the control plants (Fig. 4c) and the accumulation level of TVBMV genomic RNA in the NbPsbO1-silenced plants was about 70% lower than that in the control plants (Fig. 4d). Immunoblot analysis showed that the accumulation level of TVBMV CP in the NbPsbO1-silenced plants was significantly lower than that in the control plants (Fig. 4e).

We reported previously that TVBMV PIPOSTOP mutant could replicate normally in *N. benthamiana* leaf cells but was defective in intercellular movement^37^. In this study, we infiltrated this mutant into leaves of NbPsbO1-silenced or non-silenced control *N. benthamiana* plants. By 4 dpai, strong GFP fluorescence was observed in the infiltrated leaves of control plants. The infiltrated leaves of NbPsbO1-silenced *N. benthamiana* plants showed a very weak GFP fluorescence (Fig. 4f). Result of qRT-PCR confirmed that the accumulation level of TVBMV genomic RNA in the NbPsbO1-silenced *N. benthamiana* plants were significantly lower than that in the control plants (Fig. 4g,h). Western blotting assay agreed with the qRT-PCR result (Fig. 4i). Therefore, we concluded that NbPsbO1 played a critical role in TVBMV replication.

### Knockdown of *NbPsbO1* expression also inhibited PVY infection but not PVX

**Figure 5:**
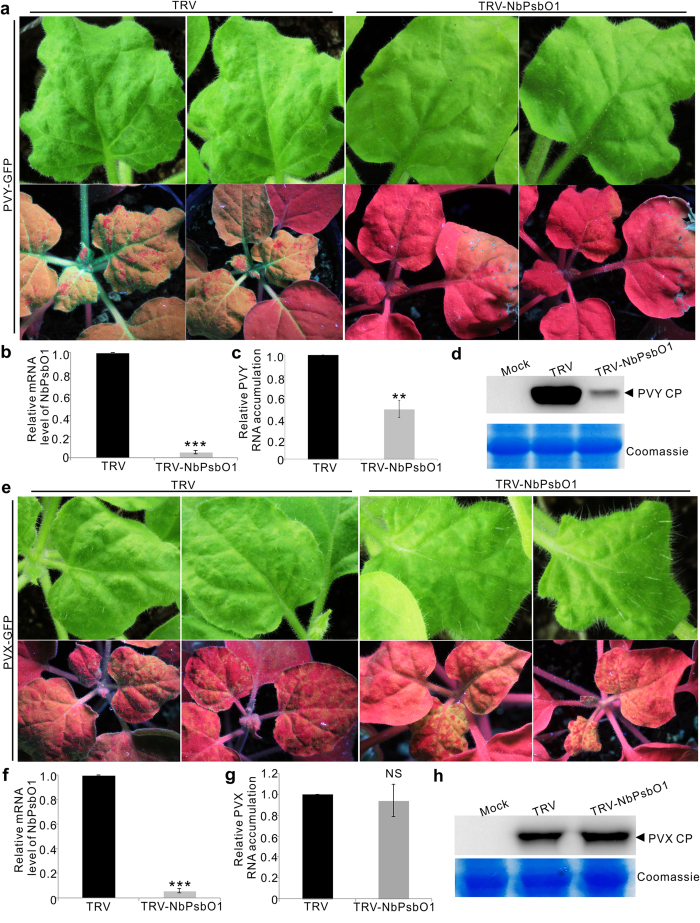
Infectivity of PVY-GFP and PVX-GFP in the NbPsbO1-silenced or non-silenced control *N. benthamiana* plants. (**a**) Virus symptoms and GFP fluorescence in PVY-GFP inoculated NbPsbO1-silenced (TRV-NbPsbO1) or non-silenced (TRV) control *N. benthamiana* plants at 10 dpi. Images were taken under the normal light (top row) or UV illumination (bottom row). (**b**,**c**) qRT-PCR analysis of *NbPsbO1* transcript and PVY genomic RNA accumulations in the NbPsbO1-silenced or non-silenced control plants. Error bars represent the standard deviations of three biological replicates. **Indicates *P* value < 0.01. ***Indicate *P* value < 0.001. (**d**) Western blot analysis for PVY CP accumulation in systemically infected leaves of the NbPsbO1-silenced and non-silenced control *N. benthamiana* plants at 10 dpi. (**e**) Virus symptoms and GFP fluorescence in the PVX-GFP inoculated NbPsbO1-silenced or non-silenced control *N. benthamiana* plants at 10 dpi. Photographs were taken under the normal light (top row) or UV illumination (bottom row). (**f**,**g**) qRT-PCR analysis of *NbPsbO1* transcript and PVX genomic RNA accumulations in the NbPsbO1-silenced or non-silenced control plants. ***Indicates *P* value < 0.001 and NS indicates no significant difference between the treatments. (**h**) Western blot analysis for PVX CP accumulation in the NbPsbO1-silenced and non-silenced plants. Coomassie Blue staining was used to show sample loadings.

To investigate the effect of NbPsbO1 on other virus infection, we rub-inoculated *Potato virus Y* (PVY)-GFP or *Potato virus X* (PVX)-GFP to NbPsbO1-silenced or non-silenced control *N. benthamiana* plants. By 10 dpi, clear mosaic symptoms and strong green fluorescence were observed in the systemically infected leaves of the control plants (Fig. 5a, left). In contrast, no mosaic symptoms and very weak green fluorescence were observed in the systemically infected leaves of the NbPsbO1-silenced plants (Fig. 5a, right). Result of qRT-PCR showed that the accumulation level of PVY genomic RNA in the NbPsbO1-silenced plants was only about half of that in the control plants (Fig. 5b,c). Similarly, the accumulation level of PVY CP in the NbPsbO1-silenced plants was significantly reduced compared with that shown by the non-silenced control plants (Fig. 5d). Interestingly, the PVX-GFP-inoculated NbPsbO1-silenced or non-silenced control plants showed similar mosaic symptoms and green fluorescence by 10 dpi (Fig. 5e). Both qRT-PCR and Western Blot assay confirmed that PVX-GFP accumulated to similar levels in both NbPsbO1-silenced and non-silenced control plants (Fig. 5g,h). These results indicated that silencing of *NbPsbO1* gene inhibited the infection of potyviruses in *N. benthamiana* plants but had no effect on the infection of PVX.

### Amino acid Asn_16_, Asp_23_ and Gly_33_ in 6K2 play important role during TVBMV replication and interaction between 6K2 and NbPsbO1

**Figure 6:**
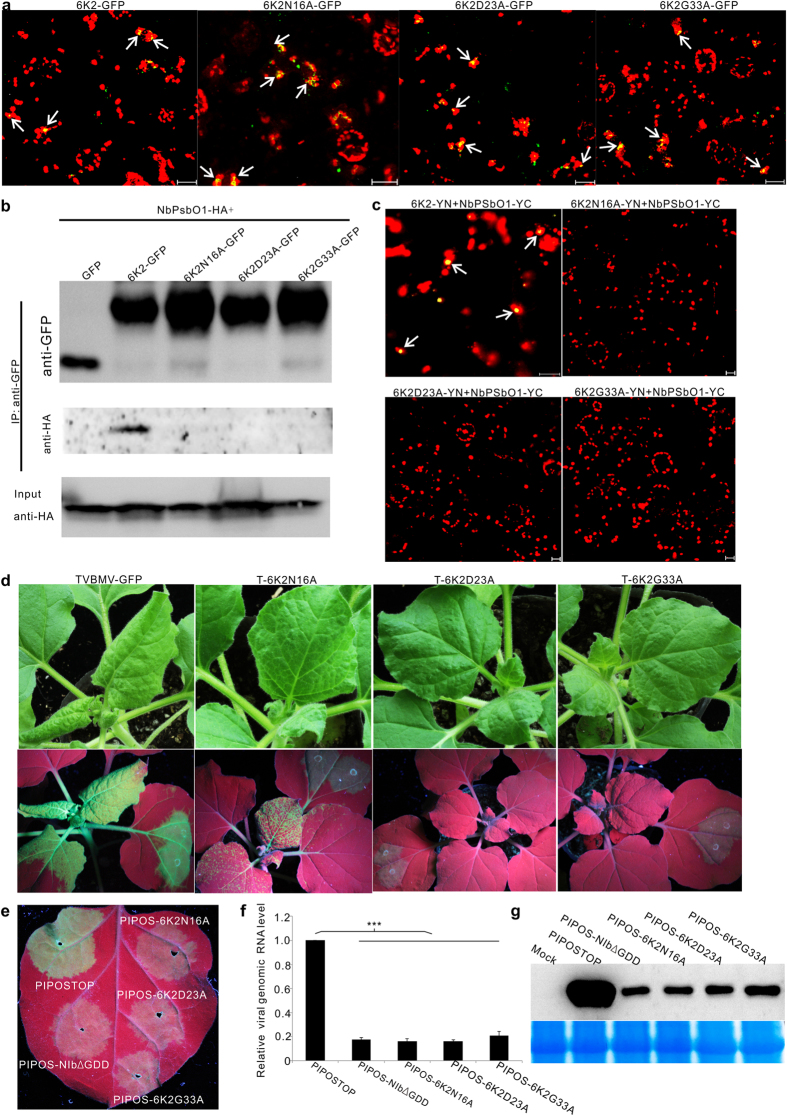
Effects of amino acid Asn16, Asp23 and Gly33 on 6K2 sub-cellular localization, interaction between 6K2 and NbPsbO1, and replication of TVBMV. (**a**) Sub-cellular localization of 6K2-GFP, 6K2N16A-GFP, 6K2D23A-GFP or 6K2G33A-GFP in *N. benthamiana* leaf epidermal cells. Arrows indicate punctates with both 6K2 green fluorescence and chloroplast red auto-fluorescence. (**b**) Analysis of interaction between NbPsbO1 and 6K2 or its mutants through Co-IP assays using an anti-GFP or an anti-HA antibody. NbPsbO1-HA and GFP-tagged 6K2 or its mutants were co-expressed in *N. benthamiana* leaves via agro-infiltration. (**c**) Analysis of interactions between NbPsbO1 and 6K2 or its mutants through BiFC assays. Images of *N. benthamiana* cells co-expressing 6K2-YN and NbPsbO1-YC, 6K2N16A-YN and NbPsbO1-YC, 6K2D23A-YN and NbPsbO1-YC, 6K2G33A-YN and NbPsbO1-YC are imaged at 48 hpai. Arrows indicate yellow fluorescent punctates. (**d**) Virus symptoms and GFP fluorescence in leaves infected with the wild-type or mutant TVBMV. Photographs were taken under the normal light or UV illumination at 7 dpai. (**e**) GFP fluorescence in the regions infiltrated with PIPOSTOP, PIPOS-6K2N16A, PIPOS-6K2D23A, PIPOS-6K2G33A or PIPOS-NIbΔGDD at 4 dpai. (**f**) qRT-PCR analysis of TVBMV genomic RNA accumulations in the PIPOSTOP or its mutants infiltrated *N. benthamiana* leaves at 4 dpai. ***Indicate *P* value < 0.001. (**g**) Western blot assay for TVBMV CP accumulation in the PIPOSTOP or its mutants infiltrated *N. benthamiana* leaves at 4 dpai.

To further investigate the interaction between 6K2 and NbPsbO1, substitution mutations were introduced into the *6K2* gene in a transient expression vector p6K2GFP to produce three mutants carrying substitution of Asn_16_ to Ala (6K2N16A-GFP), Asp_23_ to Ala (6K2D23A-GFP) or Gly_33_ to Ala (6K2G33A-GFP) in the *6K2* gene. When these three mutants were individually expressed in *N. benthamiana* leaves through agro-infiltration, they all produced green fluorescent punctates similar to that produced by the wild type 6K2-GFP in cells and most punctates were associated with chloroplast (Fig. 6a), implying that the mutations introduced into 6K2 had no effect on the subcellular distribution of 6K2. We then analyzed the interaction between individual 6K2 mutants and NbPsbO1 *in planta* via Co-IP. In this assay, GFP alone, 6K2GFP, 6K2N16A-GFP, 6K2D23A-GFP and 6K2G33A-GFP were individually co-expressed with HA-tagged NbPsbO1 in *N. benthamiana* leaves. Total protein was then extracted from the infiltrated leaves and then immunoprecipitated using GFP-Trap beads. The resulting protein samples were analyzed by Western blot assay using an HA specific antibody. The results showed that NbPsbO1 was only co-immunoprecipitated with 6K2-GFP but not the three 6K2 mutants (Fig. 6b). To confirm this finding, we conducted a BiFC assay. Our results showed that by 48 hpai, YFP fluorescence was observed in leaf cells co-expressing 6K2-YN and NbPsbO1-YC. No YFP fluorescence was observed in *N. benthamiana* leaf cells co-expressing 6K2N16A-YN and NbPsbO1-YC, 6K2D23A-YN and NbPsbO1-YC or 6K2G33A-YN and NbPsbO1-YC (Fig. 6c).

We then conducted assays to determine if these three mutations could affect TVBMV infection. The mutations were introduced individually into the pCamTVBMV-GFP infectious clone. The resulting mutant construct pCamT-6K2N16A, pCamT-6K2D23A, pCamT-6K2G33A and the parental pCamTVBMV-GFP construct were infiltrated individually into *N. benthamiana* leaves. By 7 dpai, the plants infiltrated with pCamTVBMV-GFP or pCamT-6K2N16A developed mosaic symptoms in systemically infected leaves. Under UV illumination, these plants showed GFP fluorescence in infiltrated and systemically infected leaves. It is noteworthy that the plants infiltrated with pCamT-6K2N16A showed weaker GFP fluorescence in leaves compared with the pCamTVBMV-GFP-infiltrated plants (Fig. 6d). The plants infiltrated with pCamT-6K2D23A or pCamT-6K2G33A showed GFP fluorescence in infiltrated leaves but failed to produce any virus-like symptoms and GFP fluorescence in systemically infected leaves (Fig. 6d).

We further introduced these three substitutions individually into the movement defective mutant PIPOSTOP to determine their effect on TVBMV replication. The resulting construct pCamPIPOS-6K2N16A, pCamPIPOS-6K2D23A and pCamPIPOS-6K2G33A were individually infiltrated into leaves of *N. benthamiana* plants. Plants infiltrated with pCamPIPOSTOP or pCamPIPOS-NIbΔGDD were used as controls. By 4 dpai, similar GFP fluorescence was observed in the pCamPIPOS-6K2N16A, pCamPIPOS-6K2D23A or pCamPIPOS-6K2G33A infiltrated leaves, as well as the leaves infiltrated with the replication defective mutant pCamPIPOS-NIbΔGDD (Fig. 6e). GFP fluorescence observed in these leaves were, however, weaker than that in the pCamPIPOSTOP infiltrated leaves (Fig. 6e). qRT-PCR using tissues from the infiltrated regions showed that the amount of viral genomic RNA in the pCamPIPOS-6K2N16A, pCamPIPOS-6K2D23A or pCamPIPOS-6K2G33A infiltrated leaves was similar to that in the pCamPIPOS-NIbΔGDD infiltrated leaves but significantly lower than that in the pCamPIPOSTOP infiltrated leaves (Fig. 6f). Western blot analysis showed the amount of viral CP protein in the pCamPIPOS-6K2N16A, pCamPIPOS-6K2D23A, pCamPIPOS-6K2G33A or pCamPIPOS-NIbΔGDD infiltrated leaves was similar but much lower than that in the pCamPIPOSTOP infiltrated leaves (Fig. 6g). Consequently, we concluded that amino acid Asn_16_, Asp_23_ and Gly_33_ in 6K2 were critical for TVBMV replication and the interaction with NbPsbO1.

### Photosystem II Oxygen-Evolving Complex component NbPsbP1 and NbPsbQ1 did not interact with 6K2 and did not affect TVBMV infection

**Figure 7:**
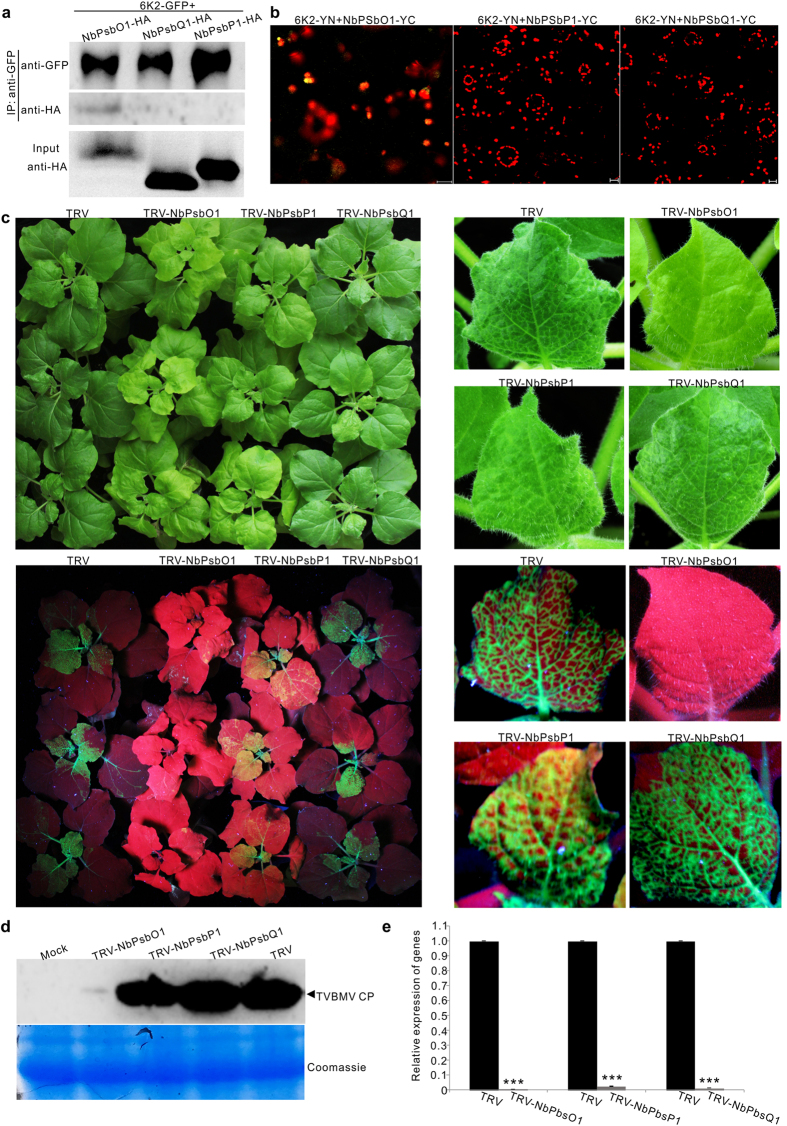
NbPsbP1 and NbPsbQ1 can neither interact with 6K2 nor regulate TVBMV infection. (**a**) Analysis of interaction between 6K2 and NbPsbP1 or NbPsbQ1 through Co-IP assays. 6K2-GFP and NbPsbO1-HA, NbPsbP1-HA or NbPsbQ1-HA were co-expressed in *N. benthamiana* leaves via agro-infiltration. Proteins were immunoprecipitated from leaf extracts with GFP-Trap beads followed by Western blot assays using a GFP- or HA-specific antibody. (**b**) BiFC assays in *N. benthamiana* leaves through co-expression of 6K2-YN and NbPsbO1-YC, 6K2-YN and NbPsbP1-YC, 6K2-YN and NbPsbQ1-YC. (**c**) Virus symptoms and GFP fluorescence in TVBMV-GFP inoculated non-silenced control (TRV), NbPsbO1-silenced (TRV-NbPsbO1), NbPsbP1-silenced (TRV-NbPsbP1) or NbPsbQ1-silenced (TRV-NbPsbQ1) plants at 6 dpi (up and low at left). A single representative leaf for each treatment is shown at right. (**d**) Western blot analysis using systemically infected leaves for TVBMV CP accumulation in TVBMV-GFP inoculated plants. Coomassie Blue staining was used to show sample loadings. (**e**) qRT-PCR analysis for *NbPsbO1, NbPsbP1* and *NbPsbQ1* transcript accumulation in gene-silenced and non-silenced control plants. Error bars represent standard deviations of three biological replicates. ***Indicate *P* value < 0.001.

PsbO, PsbP and PsbQ are three PSII extrinsic proteins in plant. To determine if 6K2 can also interact with PsbP and PsbQ, we constructed pNbPsbO1-HA, pNbPsbP1-HA and pNbPsbQ1-HA. These constructs were individually co-expressed with p6K2-GFP in *N. benthamiana* leaves. Total protein in extracts was then immunoprecipitated with GFP-Trap beads followed by Western blot assays using an HA specific antibody. The results showed that unlike NbPsbO1-HA, NbPsbP1-HA and NbPsbQ1-HA could not be pulled down by 6K2-GFP (Fig. 7a). Interaction between 6K2 and NbPsbO1, NbPsbP1 or NbPsbQ1 was further tested through BiFC assays. The results showed that by 48 hpai YFP fluorescence was not observed in leaf cells co-expressing 6K2-YN and NbPsbP1-YC or 6K2-YN and NbPsbQ1-YC. YFP fluorescence was, however, observed in the control leaf cells co-expressing 6K2-YN and NbPsbO1-YC (Fig. 7a). To determine if NbPsbP1 or NbPsbQ1 could affect TVBMV infection in plant, a 400-bp fragment representing partial *NbPsbP1* or *NbPsbQ1* sequence was inserted into the TRV-based VIGS vector and the vectors were used to silence the expression of *NbPsbP1* or *NbPsbQ1* in *N. benthamiana*. The *NbPsbO1*-silenced, *NbPsbP1*-silenced, or *NbPsbQ1*-silenced *N. benthamiana* plants were rub-inoculated with TVBMV-GFP. Plants infected with the empty TRV vector were used as controls. By 6 dpi, mosaic symptoms appeared on the TRV empty vector-infected control, *NbPsbP1*-silenced and *NbPsbQ1*-silenced *N. benthamiana* plants but no clear virus symptoms were seen in leaves of the *NbPsbO1*-silenced plants. Under UV illumination, GFP fluorescence was observed in the systemically infected leaves of the control, *NbPsbP1*-silenced and *NbPsbQ1*-silenced *N. benthamiana* plants but was not seen in the systemically infected leaves of the *NbPsbO1*-silenced plants (Fig. 7c). Immunoblot analysis using a TVBMV CP specific antibody showed that the accumulation level of TVBMV CP in the *NbPsbO1*-silenced plants was significantly lower than that from the *NbPsbP1*-silenced, *NbPsbQ1*-silenced or the control plants (Fig. 7d). Result of qRT-PCR showed that the accumulation level of *NbPsbO1, NbPsbP1* and *NbPsbQ1* in the *NbPsbO1*-silenced, *NbPsbP1*-silenced, or *NbPsbQ1*-silenced plants was only about 1.0, 2.4, and 1.3% of that from the control plants (Fig. 7e).

## Discussion

In this paper, we showed that TVBMV 6K1 and NbPsbO1, a component of the PSII Oxygen-Evolving Complex, were essential factors in the TVBMV replication complex. We also demonstrated that viral 6K1 and NbPsbO1 interacted with 6K2 and 6K1 was recruited to VRCs by 6K2. In addition, down-regulation of *NbPsbO1* expression suppressed the replication of TVBMV and PVY, but not PVX in plant.

Chloroplast is the target of many plant viruses. It was reported that several plant viruses produce VRCs adjacent to chloroplast^5^,^8^,^12^,^25^. The 6K2 protein of potyvirus was shown to induce vesicles at the ER exit site, and after exiting from ER, these vesicles trafficked to chloroplast via the early secretory pathway as well as the actomyosin system^25^. The 6K2-associated vesicles were also considered as VRCs and contained both viral RdRp and dsRNA^25^. Although 6K2 was reported to target chloroplast, no chloroplast protein has yet been demonstrated to interact with this protein. Here, we showed for the first time that TBVMV 6K1 interacted with 6K2, and was recruited to chloroplast by 6K2 (Fig. 2c,d,e). Through immunoprecipitation assay using total protein extracted from TVBMV-6K1GFP infected leaves (Supplementary Fig. 2), we identified NbPsbO1, an extrinsic OEC protein, was present in 6K1–6K2-containing VRC (Fig. 3d,f). Knockdown of *NbPsbO1* gene expression in *N. benthamiana* plants through VIGS strongly inhibited TVBMV replication in this host plant (Fig. 4). Because knockdown of *NbPsbO1* expression in *N. benthamiana* also inhibited PVY infection but not PVX (Fig. 4), we propose that NbPsbO1 is an important and specific host factor regulating potyvirus replication in plant. Although NbPsbO1 was found in the 6K1–6K2 complex, it only interacted with 6K2 *in vivo* (Fig. 3a,b). Furthermore, although NbPsbO1, NbPsbP1 and NbPsbQ1 are all components of PS II complex, TVBMV 6K2 interacted only with NbPsbO1. Knockdown of the expression of *NbPsbP1* or *NbPsbQ1* had no effect on TVBMV infection (Fig. 7). It was indicated that potyvirus utilize both chloroplast compartment and membrane for their replication^12^,^39^,^40^ (Ahlquist *et al*.^39^; Dreher^40^; Torrance *et al*.^12^). Identification of NbPsbO1 provided new evidence that potyvirus could also hijack chloroplast proteins to support their replication.

Many OEC proteins have been shown to regulate plant virus infection^17^,^18^,^19^. For example, silencing *NbPsbO* gene expression in *N. benthamiana* resulted in a significant increase of TMV, AMV and PVX genomic and subgenomic RNA accumulation in leaves^19^. Although TMV VRCs did not target chloroplast and did not co-localize with chloroplast-resident protein AtpC and RCA in cells, these two chloroplast-resident proteins did interact directly with TMV replicase based on their Co-IP assays^13^. The authors also indicated that silencing a 33-kDa OEC protein gene (*NbPsbO*) caused a general nonspecific plant response leading to an increase of multiple viruses accumulation in plant^13^. Unlike TMV, potyviruses replicate in chloroplast-bound vesicles induced by the 6K2 protein. Based on the results presented in this paper, we speculate that during potyvirus infection in plant, the 6K2 interacts with NbPsbO1 in VRCs to suppress a host defense response to allow a robust multiplication of potyvirus in plant cells. This speculation is supported by the fact that the replication of a TVBMV 6K2 mutant that has lost its ability to interact with NbPsbO1 is significantly suppressed. We also speculate that because NbPsbO1 is an important host protein for photosynthesis and is hijacked by 6K2 for virus replication, plants infected with potyviruses often showed chlorosis in leaves and stunting of the plant.

Of the 11 known potyviral proteins, the 6K1 and 6K2 are the two smallest proteins with similar molecular weight and hydrophobic motifs^41^. Positions of these two proteins have been shown to correspond to the positions of picornavirus 2B and 3A proteins that are important for virus replication and membrane binding^42^,^43^. In 2002, Kekarainen and others reported that a *Potato virus A* mutant carrying a 15-bp insert in the 5′-terminus of the *6K1* gene was non-infectious in tobacco protoplast^41^. In 2006, a 6 kDa protein of *Plum pox virus* (PPV) was identified^44^. A recent report showed that PPV 6K1 targeted VRCs during the early stage of PPV infection in plant and this 6K1 protein was an important viral factor for PPV replication in cells^31^. However, how 6K1 is recruited to VRCs remains unknown till this study. Collectively, we have now demonstrated that potyvirus 6K2 can directly interact with 6K1 and recruit 6K1 to VRCs for virus replication (Fig. 2). Through introduction of substitutions into the conserved RSD motif in 6K1, we have now confirmed that 6K1 does have a role in TVBMV replication in cells (Fig. 1b). Our results with the TVBMV cell-to-cell movement defective mutant^37^ further confirmed this conclusion (Fig. 1d–f). Because all the mutant viruses are defective in cell-to-cell movement, the difference in fluorescence intensity, RNA and CP accumulation were solely due to the decrease in replication.

**Figure 8:**
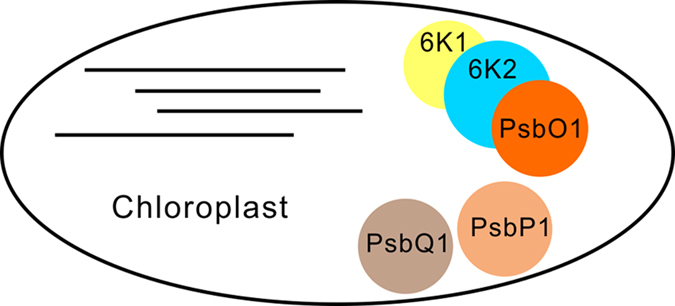
Working model for 6K2 recruiting 6K1 and hijacking PsbO1 for potyvirus replication. 6K1, 6K2 and PsbO1 are essential components of virus replication complex in association with chloroplasts. 6K2 interacts with both 6K1 and PsbO1, while 6K1 cannot interact with PsbO1 directly. 6K2 cannot interact with PsbP1 or PsbQ1. The 6K1–6K2-PsbO1 complexes regulate the replication of TVBMV and PVY in *Nicotiana benthamiana* cells.

In summary, 6K1 is a newly identified potyvirus VRCs component and is recruited to VRCs by 6K2; NbPsbO1 is a host factor present in the 6K1–6K2 complex and regulates the replication of potyviruses in cells (Fig. 8). These new findings further improve our understanding on the mechanism of potyvirus replication in plant and should provide useful hints for the development of sustainable anti-potyvirus strategies for agriculture industry.

## Methods

### Plasmid construction

TVBMV mutants were constructed through PCR-based site-directed mutagenesis described previously^37^ using specific primers listed in Supplementary Table 1. TVBMV *6K1, 6K2, CI* and *NIb* ORFs were amplified individually from pCamTVBMV-GFP^37^. Full length *NbPsbO1* was PCR amplified from cDNA derived from *N. benthamiana* leaf total RNA. The resulting PCR products were cloned individually into vector p35S::YN, p35S::YC, pCam35S::GFP or pCam35S::DsRed to generate construct p6K1-YN, p6K1-YC, p6K2-YN, p6K2-YC, pNbPsbO1-YC, p6K1-GFP, p6K1-DsRed, p6K2-GFP, pNIb-DsRed, p6K1-GFP, p6K1-DsRed, and pNbPsbO1-DsRed. All the plasmids were confirmed by sequencing before further use.

### Plant growth, protein transient expression and virus inoculation

*N. benthamiana* plants were grown inside a growth chamber set at 25 °C with 16 h light and 8 h dark cycles. Plasmid pCamTVBMV-GFP and its derivatives were introduced individually into *Agrobacterium* strain GV3101. The transformed *Agrobacterium* cells were grown overnight in the LB medium containing appropriate antibiotics and then incubated in an induction buffer [10 mM MgCl_2_, 150 μM acetosyringone and 10 mM 2-(N-Morpholino) ethane sulfonic acid (MES)] for 3 h at room temperature. Individual *Agrobacterium* culture was adjusted to OD_600_ = 0.3 for protein expression assays, OD_600_ = 0.5 for virus inoculation, or as indicated otherwise. The diluted *Agrobacterium* cultures were infiltrated into leaves of *N. benthamiana* using needleless syringes.

### Protein purification and Mass Spectrometry analysis

Total protein was extracted from 3 g *N. benthamiana* leaves infected with TVBMV-GFP or TVBMV-6K1GFP at 10 days post agro-infiltration (dpai). The crude leaf extracts were centrifuged at 20,000 *g* for 10 min, and the supernatants were incubated for 3 h at 4 °C with GFP-Trap A beads as instructed (ChromoTek, Planegg-Martinsried, Germany). The resulting protein samples were separated on 10% (w/v) SDS-PAGE gels through electrophoresis. The separated protein bands were excised and analyzed using Liquid Chromatography-Tandem Mass Spectrometry (LCTMS) by the KeeCloud Biotech Company (Shanghai, China). The LCTMS data was analyzed and then blasted against the *N. benthamiana* database at Sol Genomics Network (https://solgenomics.net/organism/Nicotiana_benthamiana/genome) with the MASCOT software.

### Confocal Microscopy and Bi-molecular Fluorescence Complementary (BiFC) Assay

For confocal microscopy, epidermal cells of agro-infiltrated *N. benthamiana* leaves were examined under a Zeiss LSM510 META laser scanning microscope or a LSM 880 Air scanning confocal microscope equipped with a Plan-Neofluar 40x/1.3 oil DIC lens or a Plan-Apochromat 63x/1.4 oil DIC lens and a multi-track mode. GFP was excited at 488 nm and the emitted signal was captured at 505 nm to 530 nm. Chloroplast auto-fluorescence was excited at 635 nm and the signal was captured after passage through a long-pass 650 nm emission filter. DsRed was excited at 543 nm and the signal was captured at 615 nm. All images were processed using the Zeiss LSM Image Examiner version 4.0 or ZEN blue version 2.1.

For BiFC assay, two fusion constructs were co-infiltrated into *N. benthamiana* leaves using needless syringes. YFP was excited at 514 nm and the signal was captured at 530–600 nm. Cells expressing the fusion proteins were imaged under the Zeiss LSM510 META laser scanning confocal microscope.

### Co-immunoprecipitation (Co-IP) and Western blot Assays

For Co-IP assays, agro-infiltrated *N. benthamiana* leaves were harvested, ground in liquid nitrogen, and homogenized in an extraction buffer as described (Geng *et al*.^37^). The crude leaf extracts were centrifuged at 20,000 *g* for 10 min and the supernatant were incubated with GFP-Trap_A beads (ChromoTek) for 3 h at 4 °C. Total protein was separated in 10% SDS-PAGE gel. After transferring protein bands to nitrocellulose membranes, the membranes were probed with a mouse anti-HA antibody (Sigma Aldrich, Shanghai, China). The detection signal was visualized using the Pierce ECL western blot substrate (Thermo Fisher Scientific, Rockford, IL, USA).

### Reverse transcription-PCR (RT-PCR) and real-time quantitative RT-PCR (qRT-PCR)

Total RNA was extracted from the harvested *N. benthamiana* leaves using Trizol reagent (TransGen Biotech, Beijing, China) followed by RNase-free DNase I treatment (New England Biolabs, Beijing, China). Reverse transcription (RT) was done with gene-specific reverse primers and the EasyScript First-Strand cDNA Synthesis SuperMix kit (TransGen Biotech). Primers used for RT-PCR were listed in Supplementary Table 1. The resulting PCR products were visualized in agarose gels through electrophoresis. qRT-PCR was performed using the UltraSYBR mixture (CWBIO, Beijing, China) and the BIO-RAD Q5 system (BIO-RAD, California, USA) as instructed. The relative expression levels of *NbPsbO1* was determined using *NbPsbO1* specific primer NbPsbO1-311F and NbPsbO1-521R (Supplementary Table 1). Relative accumulation levels of TVBMV, *Potato virus X* (PVX) and *Potato virus Y* (PVY) genomic RNA were determined using virus-specific primers (Supplementary Table 1). The accumulation level of *Elongation Factor 1a (EF-1a*) gene was determined using primer EF1a-F and EF1a-R, and was used as an internal control for the assay.

### Virus-induced gene silencing (VIGS)

Fragments covering nucleotides (nt) 463 to 962 of *NbPsbO1* (Accession no. JF897603), 73 to 472 of *NbPsbP1* (JF897607) and 76 to 465 of *NbPsbQ1* (AY887536) were selected using SGN VIGS Tool (http://vigs.solgenomics.net/) and amplified from *N. benthamiana* leaf total RNA via RT-PCR using primer pairs NbPsbO1vigs-XbaIF/ NbPsbO1vigs-BamHIR, NbPsbP1vigs-XbaIF/ NbPsbP1vigs-BamHIR and NbPsbQ1vigs-XbaIF/ NbPsbQ1vigs-BamHIR, respectively. The resulting PCR products were cloned individually into the pTRV2 vector^38^ to generate pTRV2-NbPsbO1, pTRV2-NbPsbP1 and pTRV2-NbPsbQ1. *Agrobacterium* cultures harboring pTRV1, pTRV2 or pTRV2 derivatives were grown individually overnight in LB medium containing appropriate antibiotics, pelleted and then incubated in the induction buffer for 3 h at room temperature. *Agrobacterium* culture harboring pTRV1 was mixed with an equal volume of *Agrobacterium* culture harboring pTRV2 or one of the three pTRV2 derivatives. The mixed *Agrobacterium* cultures were infiltrated individually into *N. benthamiana* leaves. At 14 dpai, the upper non-infiltrated *N. benthamiana* leaves were inoculated with crude extracts from TVBMV-GFP, PVY-GFP or PVX-GFP-infected *N. benthamiana* leaves.

## Additional Information

**How to cite this article**: Geng, C. *et al*. *Tobacco vein banding mosaic virus* 6K2 Protein Hijacks NbPsbO1 for Virus Replication. *Sci. Rep.* **7**, 43455; doi: 10.1038/srep43455 (2017).

**Publisher's note:** Springer Nature remains neutral with regard to jurisdictional claims in published maps and institutional affiliations.

## Supplementary Material

Supplementary Information
