# Supplementary material for: Tobacco vein banding mosaic virus 6K2 Protein Hijacks NbPsbO1 for Virus Replication
Source: Sci Rep. 2017 Feb 23;7:43455. doi: 10.1038/srep43455 (PMC5322494; doi:10.1038/srep43455)
Supplement: Supplementary Information [file srep43455-s1.pdf]

Tobacco vein banding mosaic virus 6K2 Protein Hijacks NbPsbO1 for Virus Replication

Chao Geng[1], Zhi-Yong Yan[1], De-Jie Cheng[1], Jin Liu[1], Yan-Ping Tian[1], Chang-Xiang Zhu[1, 2], Hong-Yan Wang[1] & Xiang-Dong Li[1, 2]

[1]Laboratory of Plant Virology, Department of Plant Pathology, College of Plant Protection, Shandong Agricultural University, Tai’an, Shandong 271018, China. [2]Shandong Provincial Key laboratory for Agricultural Microbiology, Tai’an, Shandong, 271018, China. Correspondence and requests for materials should be addressed to X.L. (email: xdongli@sdau.edu.cn)

Supplementary information

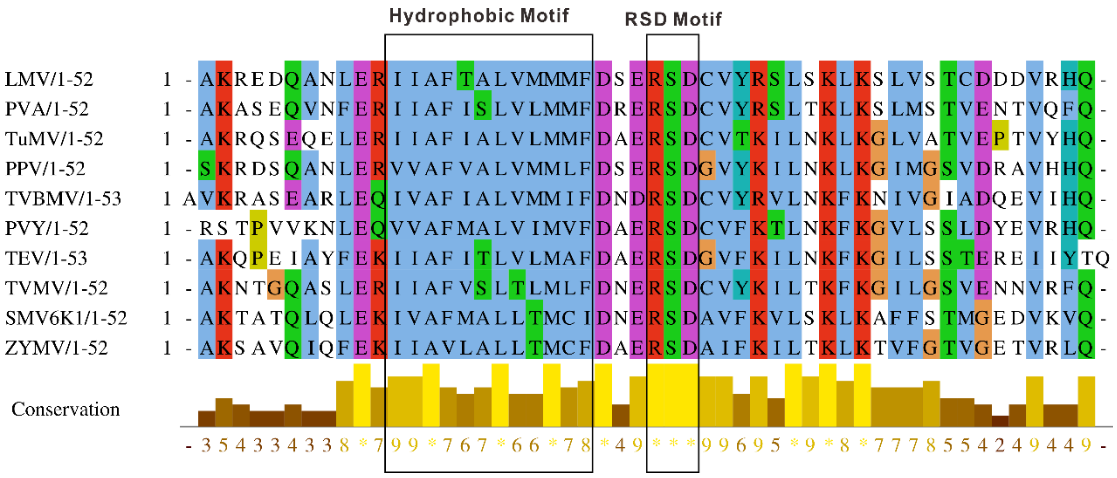

Supplementary Figure S1. Amino acid alignment using ten potyvirus 6K1 proteins. The amino acid color coding is done according to the Clustal X color scheme.

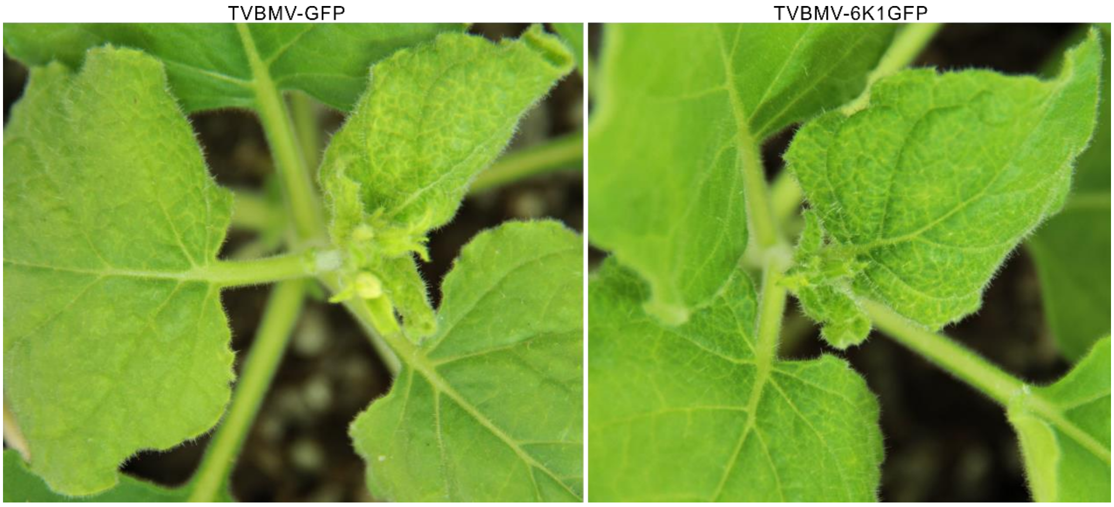

Supplementary Figure S2 Infection of TVMBV-GPP and TVBMV-6K1GFP in *N. benthamiana* plants. The TVBMV-GFP and TVBMV-6K1GFP inoculated *N. benthamiana* plants were photographed at 7 dpai.

Supplementary Table S1

Primer Name	Primer Sequence
T-6K1ASD-F	TGACAATGATGCGAGTGATTGTGTCTATAGAGTTTTAAACAA ATTTAAGAACATAG
T-6K1ASD-R	ATCACTCGCATCATTGTCAAAGATCATCATCACCAGAGCTAT AAATG
T-6K1RSA-F	TGACAATGATAGGAGTGCATGTGTCTATAGAGTTTTAAACAA ATTTAAGAACATAG
T-6K1RSA-R	TGCACTCCTATCATTGTCAAAGATCATCATCACCAGAGCTAT AAATG
T-6K1RAD-F	TGACAATGATAGGGCTGATTGTGTCTATAGAGTTTTAAACAA ATTTAAGAACATAG
T-6K1RAD-R	ATCAGCCCTATCATTGTCAAAGATCATCATCACCAGAGCTAT AAATG
NbPsbO1-XbaIF	GCTCTAGAATGGCTGTCTCTTTACAAGCAG
NbPsbO1BamHI R	CGGGATCCTTCAAGTTGGGCATACCAG
T-6K1-XbaI-F	GCTCTAGAATGGCTGTAAAGCGAGCATCGG
T-6K1-BamHIR	CGGGATCCTTGGTGAATCACCTCTTGG
T-6K2-XbaI-F	GCTCTAGAATGAGTAAGGACGAAATTGCAG
T-6K2-BamHIR	CGGGATCCTTGATGTTCAACAACCTC
T-CPqPCR130-F	GGCACATTCTCAATACCG
T-CPqPCR318-R	GTCATAATCGCCCTTCAC
EF1α-F	TGGTGTCCTCAAGCCTGGTAT

EF1α-R	ACGCTTGAGATCCTTAACCGC
NbPsbO1vigs-XbaIF	GCTCTAGACCCCAGATTTCCAGAAAACTAAG
NbPsbO1vigsBamHIR	CGGGATCCCACATCCTTGGGGACCTTTG
NbPsbO1-311-F	AAGTAAAGGGAACTGGAAC
NbPsbO1-521-R	ATCTCATCAAGGGTGTAGG
PVY-CP-418qPCRF	CAAGTCGAATACCCACTGAAACC
PVY-CP-722qPCRR	GTACTAATGCCACCATCCAATCC
PVX1985-CP490qPCRF	TTCTTCAACGGAGTCACCAACCC
PVX1985-C613qPCRR	CCCTGGCCTTCGTAATCTTCACA
NbPSbP1vigs-XbaI	GCTCTAGAATTGTTCCATCACTCAAGCC
NbPSbP1vigs-BamHIR	CGGGATCCGTTCAGGGGAACCAAAGTCAG
NbPSbQ1vigs-XbaI	GCTCTAGATCAGCCCGTTTGAGCACTGT
NbPSbQ1vigs-BamHIR	CGGGATCCGACGAAGGTCATTCTGGACATATG
NbPsbP42-qPCRF	AACTCCAGCTAGAACTCCCTTAG
NbPsbP317-qPCRR	TTGAATCCATCTCCGTTGTATGT
NbPsbQ5-qPCRF	CTCATGCTATGGCTTCAATGGGTG
NbPsbQ104-qPCRR	CTGGTGCTAACAGTGCTCAAACG
T-6K2N16A-MF56.5	CGTTGGGCTAAATCTCTAATCACACGGGATTTGTTAGTTTGTTTTG
T-6K2N16A-MR64	ATTAGAGATTTAGCCCAACGACTCTTGAGTTTGAGTGCTGCTG
T-6K2D23A-MF58.5	ACACGGGCATTGTTAGTTTGTTTTGGCGTGGCAGCTG
T-6K2D23A-MR66	AAACTAACAATGCCCGTGTGATTAGAGATTTGTTCCAACGACTCTTG
T-6K2G33A-MF63.5	AGCTGCTGGTGCATGGATGATATACCAATATTTGATGGACAGATGCAATG
T-6K2G33A-MR68.5	ATCCATGCACCAGCAGCTGCCACGCCAAAACAAACTAACAAATC

T-NIbGDDMF	CGCTTTCGTTGCAAATCTCATTATTGCAGTGGCACCAGGGAG TG
T-NIbGDDMR	ATGAGATTTGCAACGAAAGCGCAATGCTCTGAGAAATTCTCA TAAGAAATCCCT
NbPsbO1-XbaIF	GCTCTAGAATGGCTGTCTCTTTACAAGCAG
NbPSbO1HA-BamHIR	CGGGATCCAGCGTAATCTGGAACATCGTATGGGTATTCAAGT TGGGCATACCAG
NbPSbP1XbaI	GCTCTAGAATGGCTTCCACACAATGCTTC
NbPSbP1HA-BamHIR	CGGGATCCAGCAAGGCTGAAAGAAGTGACAG
NbPSbQ1XbaI	GCTCTAGAATGGCTCATGCTATGGCTTCAATG
NbPSbQ1HA-BamHIR	CGGGATCCACCAAGTTTGGCCAAAACATC
NbPSbP1-BamHIR	CGGGATCCAGCAAGGCTGAAAGAAGTGACAG
NbPSbQ1-BamHIR	CGGGATCCACCAAGTTTGGCCAAAACATC

T, TVBMV; F, forward; R, reverse; M, mutation.
